# Evidence-based exercise recommendations to improve functional mobility in older adults - A study protocol for living systematic review and meta-analysis

**DOI:** 10.12688/openreseurope.17823.1

**Published:** 2024-09-11

**Authors:** Bettina Wollesen, Tamar Yellon, Antoine Langeard, Vera Belkin, Anna Wunderlich, Eleftheria Giannouli, Guoping Qian, Rafael A. Bernades, Zbigniew Ossowski, Uros Marusic, Rajesh Sighdel, Yael Netz, Claudia Volecker-Rehage

**Affiliations:** 1 Human Movement Science, Universitat Hamburg, Hamburg, Hamburg, 20148, Germany; 2 Henrietta Szold Hadassah Hebrew University School of Nursing, University Jerusalem, Jerusalem, Israel; 3 COMETE UMR-S 1075, GIP Cyceron, Université de Caen Normandie, INSERM, Normandie Université, GIP Cyceron, Caen, Normandie, France, Caen, Normandie, France; 4 Neuromotor Behavior and Exercise, University of Münster, Münster, Germany; 5 Biological Psychology and Neuroergonomics, Technische Universitat Berlin, Berlin, Berlin, Germany; 6 Department of Health Sciences and Technology, ETH Zurich, Zurich, Switzerland; 7 Faculty of Physical Culture, Gdansk University of Physical Education and Sport, Gdansk, Poland; 8 Center for Interdisciplinary Research in Health Sciences, Faculty of Health Sciences and Nurses, Universidade Catolica Portuguesa, Lisbon, Portugal; 9 Institute for Kinesiology Research, Science and Research Centre Koper, Koper, Slovenia; 10 Department of Health Sciences, Alma Mater Europaea University, Maribor, Slovenia; 11 Wingate Campus, The Levinsky-Wingate Academic College, Netanya, Israel; 12 Department of Health Promotion and Rehabilitation, Lithuanian Sports University, Lithuanian Sports University, Kaunas, Lithuania

**Keywords:** functional mobility, older adults, physical training interventions, evidence-based exercise

## Abstract

**Background and objectives:**

This is a protocol for a living systematic review and meta-analysis.

This review will assess the effects of state-of-the-art exercise interventions designed to promote mobility. Therefore, after identifying all potential interventions, we will use the F.I.T.T. principles as well as the physical and health status of the participants as moderators to analyse the mechanisms for the positive benefits of exercise interventions.

The main research questions are:

- Which exercise types are most beneficial for improving functional mobility in various populations of older adults?
- Which physical exercise characteristics in terms of frequency, intensity, time and duration will achieve the greatest benefit in terms of the defined outcomes, i.e, the functional mobility of older adults?

**Methods:**

The systematic literature research according to PRISMA guidelines will search databases like MEDLINE, APA Psych-Info and Web of Science.

Inclusion criteria are: healthy older people ≥ 50 years, randomized-controlled trials including exercise intervention and a walking or mobility assessments (eg., TUG, SPPB) as an outcome measure. A preliminary search revealed more than 33,000 hits that will be screened by pairs of independent reviewers. The results will be summarized according to the effects regarding functional mobility and potential dose-response relations via respective meta-analysis.

**Conclusion:**

The systematic review will comprise the knowledge of the existing literature with regards to the effects of the physical activity interventions compared to an active or inactive control group.

We will summarize the effects with respect to the F.I.T.T principles. If possible, we will also compare interventions from the different categories (cardiovascular exercise, resistance exercise, motor-coordinative exercise, multicomponent exercise, mind-body exercise, exergames, hybrid exercise, and concurrent training) as a network analysis and report the influence of moderator variables. Based on the results evidence-based guidelines following GRADE for physical exercise interventions to improve mobility in older adults will be provided.

## Introduction

The increasing life expectancy of older adults is coupled with a rising number of older adults needing support for (instrumental) activities of daily living and/or living in nursing homes ( England & Azzopardi-Muscat, 2017). Factors of dependency and morbidity in old age have substantial impacts on public health systems worldwide, primarily due to escalating healthcare costs ( Albert *et al.*, 2015). Functional mobility of older adults is a crucial determinant of health and independence ( Braun *et al.*, 2022). Mobility can reverse or delay age-related health issues and promote independence, while immobility or limitations in mobility (such as difficulty rising from a chair, balance problems, or reduced walking capacity) can cause or increase dependency. This underscores the significance of fostering mobility among the growing population of older adults. Moreover, in some settings (eg.in stationary or ambulatory care) older adults are a vulnerable population with an increased risk of discrimination and exclusion from society, partly due to unmet mobility needs ( Nanninga *et al.*, 2018; Van Biljon *et al.*, 2022). The development of tailored exercise programs targeting the independence of older adults is one of the motivators for the improvement of crucial policy action areas described by WHO in the Equity Policy Tool, namely *ʻliving conditionsʼ* and *ʻsocial and human capitalʼ* ( WHO, 2019)

Exercise can be seen as a preventive measure, so that, without adequate physical activity levels, the ageing process may be associated with premature development of disease and dysfunction ( Izquierdo *et al.*, 2021).

### Mobility in older adults

According to the World Health Organization's (WHO) International Classification of Functioning (ICF) model, overall mobility comprises activities such as “moving by changing body position or location or by transferring from one place to another by carrying, moving, or manipulating objects, by walking, running, or climbing, and by using various forms of transportation” ( Liu, 2017). The ICF highlights the interdependence of activities with body functions and structures, participation, and personal and environmental factors for overall mobility.

The key outcomes related to functional mobility in older age include gait, walking capacity, and balance. Additionally, aspects of life-space mobility, such as the overall walking distance within the environment, are significant factors that characterize mobility in older adults. These outcomes were rated as highly important in the Delphi study on mobility ( Vogel *et al.*, 2023). Moreover, functional mobility deficits have important clinical implications, as the magnitude of these deficits significantly influences the prognosis and benefits of certain interventional therapies ( Pavasini *et al.*, 2016). Functional mobility is assessed by different outcome measures, and these parameters have been associated with different health outcomes.

***Walking capacity and gait performance***

Walking capacity and gait performance are components of functional mobility and affect the overall mobility of older adults. Decreased walking capacity (e.g., expressed by the 6-minute walking test (6MWT or the ability to walk > 400 m) as well as a rapid age-related decline in gait speed, are associated with increased mortality ( Cesari *et al.*, 2005; Ferrucci & Guralnik, 2013; Studenski *et al.*, 2011; Van Kan *et al.*, 2009; White *et al.*, 2013), social isolation, and dependency ( Bevilacqua *et al.*, 2021; Hinrichs *et al.*, 2022). Conversely, improvement in gait speed predicts an increased lifespan ( Hardy *et al.*, 2007). Moreover, to navigate in daily traffic situations, a walking speed of 1.2 m/s is required to cross the street during the green phase of traffic lights ( Donoghue *et al.*, 2016). In addition to walking capacity and gait speed, gait performance is assessed by use of gait parameters of rhythm and phase (e.g., “stance time”, “single support time”, “double support time,” and cadence), as well as gait variability (for an overview cf. Beauchet *et al.*, 2017).

***Short Physical Performance Battery (SPPB) and Timed Up and Go (TUG)***

The risk of inability to cope with everyday life situations increases with age. The reasons for this vary. However, the clinical picture is typically consistent, including mobility, strength, and balance problems ( Fried *et al.*, 2001; Pavasini *et al.*, 2016). There are many assessments for measuring the extent of these deficits and the degree to which a person’s independence is limited. Two of the most frequently used assessments are the Timed Up and Go (TUG) ( Podsiadlo & Richardson, 1991) and the Short Physical Performance Battery (SPPB) ( Guralnik *et al.*, 1994). Due to their practicability and validity, they are particularly recommended for assessing functional mobility in older adults ( Vogel *et al.*, 2023). The TUG is also recommended as a routine screening test for the examination of basic everyday movements and daily life tasks (standing, walking, and turning; Soubra *et al.*, 2019). The SPPB is used to examine gait, balance, and strength in older adults. SPPB scores are predictors of a wide range of health outcomes, such as the disability score in Activities of Daily Living (ADLs), loss of mobility, and future hospitalization ( Gómez *et al.*, 2013). Moreover, recent studies have shown that the SPPB total score was independently associated with reported falls in older outpatients ( Lauretani *et al.*, 2019).

***Physical Activity (PA) level***

In addition to the individuals´ gait and balance performance, mobility is often described by the level of PA. To avoid biased self-reported methods of assessing PA, accelerometers have been used as a valid objective measure. Besides PA level, accelerometers can be used to assess posture, posture changes, and estimated energy expenditure ( Yang & Hsu, 2010). Devices differ in the number and type of gathered parameters, captured movement axis, and reported metrics, complicating the comparability of results. In general, a measurement period of seven days and 10 hours each day is recommended to reliably capture habitual physical activity ( Gabrys *et al.*, 2015).

### Exercise interventions to increase mobility in older adults

It has been shown that different types of exercise such as resistance and aerobic exercises (or cardiovascular exercise), especially multicomponent training, act as stimulants to functional mobility ( Da Silveira Langoni *et al.*, 2019). For example, a multicomponent exercise program with progressive, individualized resistance tasks, coupled with walking and balance activities, is helpful to preserve or improve physical determinants of mobility, particularly strength, balance and flexibility ( Brahms *et al.*, 2021; Sáez de Asteasu *et al.*, 2020). With respect to gait performance, a previous meta-analysis compared specific training modalities (strength/power, coordination, and multimodal exercise training) ( Hortobágyi *et al.*, 2015). It demonstrated the efficacy of commonly used interventions for the improvement of habitual and increased gait speed in significant and clinically relevant ways for healthy older adults aged 65 and older ( Hortobágyi *et al.*, 2015). Interestingly, the authors considered the number of included studies as relatively low and mentioned the need for an update as soon as an adequate number of studies are available.

Moreover, there is some evidence that exercise interventions positively affect other functional mobility outcomes, such as balance assessed by the SPPB and/or the Timed Up and Go test ( Chou *et al.*, 2012; Falck *et al.*, 2019; Hortobágyi *et al.*, 2015; Zhang *et al.*, 2020). The systematic review by Cordes *et al.* (2021) compared different chair-based exercise interventions for nursing home residents. They found that a multicomponent strength (or resistance exercise) and flexibility program with ankle and wrist weights ( Baum *et al.*, 2003) as well as a seated range of motions (ROM) training with ROM exercises for the fingers, hands, arms, and legs and low intensity physical activity ( Lazowski *et al.*, 1999) increased the TUG score in correlation to increased physical functions. In addition, a high-speed resistance exercise training on cognitive function and physical performance twice a week for one hour over a 16-week intervention period conducted by Yoon, Lee, & Song (2018) improved the SPPB in older adults.

A recent review contextualized physical exercise benefits and the individual mobility status with respect to physical fitness (walking capacity) at baseline. Older adults with low walking capacity (i.e., walking speed <0.8 (m·s−1)) appear to benefit most from an untargeted intervention, while older adults with more preserved walking capacity probably benefit most from specific balance and strength training ( Brahms *et al.*, 2021). However, this model still requires additional verification. Moreover, motor-coordinative exercises, considered those that focus on improving motor skills, coordination, balance, and agility have been shown to have specific benefits on motor performance in older adults ( Hübner & Voelcker-Rehage, 2017). These exercises often involve complex movements that challenge the neuromuscular system and improve coordination between muscles and nerves. Examples include agility drills, balance exercises (e.g., standing on one leg), and sports-specific drills (e.g., dribbling in basketball, footwork in soccer).

Current exercise healthcare trends include mind-body exercises, which integrate physical movement with mental focus and relaxation techniques to promote overall well-being. These exercises aim to improve mind-body connection, reduce stress, and enhance mental clarity and emotional balance. Examples include yoga, tai chi, qigong, and Pilates. Studies have highlighted their benefits on overall coordination and cognitive function ( Blomstrand *et al.*, 2023). Furthermore, exergames might also be used on contemporary exercise programs. They typically use motion-sensing technology, such as motion controllers or cameras, to track players' movements and translate them into in-game actions. Exergames have a great potential to improve cognitive-motor performance in older adults ( Gallou-Guyot *et al.*, 2020).

Moreover, different training types will enhance outcomes of functional mobility via specific pathways. For example, walking capacity has an endurance and a strength component. Accordingly, aerobic training will improve walking capacity by enhancing cardio-respiratory fitness, as shown within different clinical trials with older patient cohorts ( MacKay-Lyons *et al.*, 2020). Resistance training helps to increase muscle strength in the lower limb, which increases gait stability ( Brach & Van Swearingen, 2013; Fragala *et al.*, 2019). However, recent literature reviews showed that a combination of both interventions might be more sufficient to gain most benefits on walking capacity ( Lee & Stone, 2020).

In addition, gait performance, evaluated through the different gait parameters previously mentioned, relies on numerous physiological functions which undergo age-related declines. Mechanisms through which physical exercise training can improve gait performances in older adults are related to the effect of the different types of exercises on these physiological functions: proprioception can be improved by balance exercises, motor control can be improved by coordination exercises, muscular function can be improved by strength exercises ( Hortobágyi *et al.*, 2015; Lopopolo *et al.*, 2006). Among those, increasing muscle strength may be the most beneficial type of exercise to improve gait speed ( Hortobágyi *et al.*, 2015). Regardless of exercise type, higher intensity, frequency and duration are shown to have greater effects on gait speed ( Lopopolo *et al.*, 2006). Moreover, there is a lot of evidence, that cognitive-motor exercise can improve gait parameters in different target groups of older adults (eg., with hearing impairments, Parkinsons disease, with a history of falls ( De Freitas *et al.*, 2020; Fritz *et al.*, 2015; Wollesen *et al.*, 2022). The effects of these interventions are explained by brain-body facilitation processes that help to coordinate motor control ( Herold *et al.*, 2018).

Improved walking capacity and gait stability via different exercise forms might further lead to increased physical activity with higher distances of walking or other forms of mobility in the environment (eg., increased life space mobility, Johnson *et al.*, 2020). This increase of activity caused by functional mobility can be assessed by the number of steps per day or lower energy costs ( Dunlap *et al.*, 2022).

Finally, relevant training parameters are often defined using the F.I.T.T. principles ( Garber *et al.*, 2011). These include Frequency, Intensity, Time (duration), and Type (form) of training. According to evidence-based training principles and control mechanisms, physical training should be designed so that it is specific to the aim of the training, tailored to the individual preconditions and needs, induce an adequate overload of physiological adaptation, and be designed progressively to continuously adjust the load to the individual’s performance (progressive challenge) ( Hecksteden *et al.*, 2018; Herold *et al.* 2019). Training progression could be achieved by increasing the training load (e.g., higher number of repetitions or training sets, walking distances, and prolonged training duration), the training frequency (higher number of training units per week), and/or the training intensity (additional weights and more difficult exercise variants). All these aspects are considered as important prerequisits for muscular and cardiovascular adaptation/improvement through exercise interventions (exercise interventions ( Chodzko-Zajko *et al.*, 2009; Jahanpeyma *et al.*, 2021). The assessment of these training principles and training control mechanisms helps to provide evidence of the effectiveness of different training interventions and their composition (dose-response relationships). Thus, in order to conduct a systematic review and meta-analysis aiming to develop evidence-based guidelines for physical exercise to improve mobility in older adults, it is necessary to comprehensively assess the exercise principles. This review will only consider studies that report/describe the training intervention according to FITT principles for their interventions and delivery modalities.

## Objectives

This review will systematically assess the effects of state-of-the-art exercise interventions designed to promote mobility. Therefore, after identifying all potential interventions, we will use the F.I.T.T. principles as well as the physical and health status of the participants as moderators to analyse the mechanisms for the positive benefits of exercise interventions. The ultimate aim is to provide evidence-based guidelines for the optimal type and dose of exercise to preserve and enhance functional mobility in diverse populations of older adults. A comprehensive network and moderator metanalysis is needed to answer the following research questions:

- Which exercise types are most beneficial for improving functional mobility in various populations of older adults?
- Which physical exercise characteristics in terms of frequency, intensity, time and duration will achieve the greatest benefit in terms of the defined outcomes, i.e the functional mobility of older adults?

## Methods

### Study design

This living systematic review aims to address the broad and holistic exploration that is required to answer the research questions. To design, review, and report this review, we will use the Preferred Reporting Items for Systematic Reviews and Meta-Analysis extensions protocol (PRISMA-P; Moher *et al.*, 2015).

### Eligibility criteria

The following section describes the eligibility criteria according to the PICO(S) scheme.

### Types of studies

The review will include randomized controlled trials (RCTs), considering both published and unpublished sources. This will include randomized cross-over, cluster, and parallel-arm trials. All interventions competing in the review will be eligible for randomization together. Quasi-randomized studies will be excluded from consideration.

The search will span all eligible studies across different languages accessible through databases ( Lefebvre *et al.*, 2019). Only studies reporting trial results will be incorporated. In cases where multiple studies cover the same or overlapping populations, priority will be given to the study with the highest participant count or the one following pre-registration in a trial register.

Reference lists from included studies and relevant systematic reviews will be examined. Additionally, efforts will be made to identify ongoing and unpublished trials by contacting researchers and organizations in the field. This will involve checking clinical trial registers such as ClinicalTrials.gov, the International Clinical Trials Registry Platform (ICTRP), or the German Clinical Trials Register (DRKS). Any missing data from RCTs will also prompt further investigation.

To maximize relevant evidence, grey literature sources like reports, dissertations, theses, and conference abstracts will be searched comprehensively.

### Types of participants

We will include studies involving participants aged > 60, according to the WHO definition of "older adults" (mean age ± SD, with a minimum age of 50). This will cover older individuals from various settings, including community-dwelling individuals, those residing in residential homes, nursing homes, assisted living facilities, retirement communities, and similar environments. Exclusions will be applied to trials focusing exclusively on specific conditions or medical diagnoses (e.g., rheumatoid arthritis, stroke), as well as studies reporting interventions exclusively aimed at inpatient rehabilitation (e.g., post-surgery care).

In addressing studies with a mixture of eligible and non-eligible participants, we will adopt a systematic approach during screening.

Firstly, we will meticulously assess each study's eligibility criteria to ascertain if they meet our age inclusion (> mean of 60 years) and exclusion criteria for specific conditions. Studies clearly indicate inclusion of participants within our age range and not exclusively focused on excluded conditions will be deemed eligible.

For studies including both eligible and non-eligible participants, we will extract data solely from eligible participants. If needed, we will contact study authors for clarification on participant demographics and eligibility.

During data synthesis and analysis, we will ensure appropriate interpretation and reporting of findings from studies with mixed participant populations. This may involve subgroup or sensitivity analyses to gauge the impact of including non-eligible participants on our review's overall outcomes and conclusions.

### Types of interventions

The descriptions of interventions used in individual trials will be examined and the exercise interventions will be categorized according to the definition of the American College of Sports Medicine ( Swain *et al.*, 2014) as any kind of "planned, structured, and repetitive bodily movement done to improve or maintain one or more components of physical fitness". This can be cardiovascular exercise, resistance exercise, motor-coordinative exercise, multicomponent exercise, mind-body exercise, exergames, combined exercise (e.g., the combination of aerobic and resistance exercises) and concurrent training (e.g., cognitive and motor training simultaneously). The frequency, duration and type of exercise has to be defined/reported within the study. Studies assessing the effect of acute (single session) exercise will be excluded.

For our main comparisons, we will include trials where the intervention was compared with 'usual care' (i.e., no change in usual activities) or a control intervention (i.e., waiting list, stretching, health education recreational activities). Treatment as usual includes for example classical therapy for the diagnosed pathology in the control group.

In our review, some studies may meet certain inclusion criteria but not others. For example, a study may meet the criteria for intervention type and comparator but may lack clear reporting on exercise parameters such as frequency, duration, and type. In such cases, these studies may still be included in the overall analysis but may be excluded from specific analysis steps within the moderator analysis. However, the corresponding authors will be asked for additional information, to ensure that all eligible studies can be integrated into the analysis. This approach is to ensure consistency and rigor in the analysis while maximizing the inclusion of relevant studies. By including these studies in the overall analysis, we maintain a comprehensive assessment of the available evidence. However, for specific analysis steps within the moderator analysis where clear and consistent reporting of certain criteria is crucial, such as subgroup analyses based on exercise parameters, studies lacking this information may not provide meaningful data and could potentially introduce bias or confound the analysis. Therefore, excluding these studies from specific analysis steps within the moderator analysis helps to ensure the robustness and validity of the results.

For this review, ‘'classical therapy’ refers to traditional or conventional forms of therapy or treatment that have been established and used for a significant period, which might differ considering settings or pathologies. It might be standard pharmacological treatments or established medical procedures. In rehabilitation, it usually consists of conventional exercise programs, manual therapy techniques, or standard rehabilitation protocols commonly used for a particular condition or injury.

Acute exercise studies (studies assessing the effect of acute (single session)) will be excluded. 

### Types of outcome measures

We will collect data from studies that report baseline assessments and post assessments. If multiple post assessments are available, we will use the one closest to the end of the exercise intervention for the meta-analysis. Nevertheless, we will report results of follow-up measurements to describe maintaining effects. Clinically meaningful timing for outcome assessments will be dependent on the type of program applied and pathology under treatment. In this sense, our review will consider short-term assessments (in the first six months of treatment) and between six to 12 months of treatment.

### Primary outcomes

Parameters representing functional mobility ( *The Short Physical Performance Battery* (SPPB) ( Guralnik *et al.*, 1994); *Timed up and Go* test (TUG) ( Podsiadlo & Richardson, 1991), i.e., the standard or mobile assessment of the TUG ( Herman *et al.*, 2011); *6minute walk test* 6MWT ( Sciurba & Slivka, 1998) for walking capacity; *Walking distance and number of steps per day (*according to accelerometer dat *a)* will be assessed as primary outcomes to evaluate the intervention effects.

### Secondary outcomes

The secondary outcomes include additional *Gait parameters.* Selection of gait parameters is based on a factor analysis by Hollman *et al.* (2011) that identified five domains of gait performance (see Table 1) and the Canadian Guidelines ( Beauchet *et al.*, 2017) for the assessment of gait parameters (see Table 1 for an overview of all parameters).

**Table 1.** Summary of the recommended gait parameters.

Domains ( Hollman *et al.*, 2011)	Parameters
Rhythm (steps/min; s)	Cadence, step time ( Hollman *et al.*, 2011) stride time, swing time, *stance time*, *single support time* ( Beauchet *et al.*, 2017; Hollman *et al.*, 2011)
Phase (%GC)	temporophasic parameters swing and stance ( Hollman *et al.*, 2011) *single* *and double support, double support time* ( Beauchet *et al.*, 2017; Hollman *et al.*, 2011)
Variablility (%CV)	gait cycle, step variability parameters step length and step time ( Hollman *et al.*, 2011) *stride length*, stride time, swing time, *stance time*, *stride speed* ( Beauchet *et al.*, 2017; Hollman *et al.*, 2011)
Pace (cm/s; cm)	*gait speed* and *stride length* ( Beauchet *et al.*, 2017; Hollman *et al.*, 2011) step length ( Beauchet *et al.*, 2017)
Base of support (cm)	step width ( Beauchet *et al.*, 2017; Hollman *et al.*, 2011) step width variability ( Hollman *et al.*, 2011)

Adapted by Beauchet *et al.*, 2017; Hollman *et al.*, 2011.

The review will also include the following adverse effect outcomes reported within the included studies:

- Musculoskeletal injuries: older adults may be at increased risk of musculoskeletal injuries such as strains, sprains, or fractures due to factors like reduced bone density, muscle mass, and joint flexibility. Exercises with high impact or high intensity, improper form, or inadequate warm-up may increase the risk of these injuries.
- Falls and Balance Issues: certain exercises that challenge balance or involve rapid movements may increase the risk of falls, especially in older adults with balance impairments or mobility issues. Falls can lead to serious injuries such as fractures, head trauma, or lacerations.
- Exacerbation of pre-existing conditions: exercise interventions that are not tailored to individual health status or medical conditions may exacerbate pre-existing health issues such as arthritis, osteoporosis, or chronic pain. For example, high-impact exercises may worsen joint pain in individuals with arthritis.

### Information sources

Literature searches will mainly be conducted in electronic databases: Medline (1946 to current), Embase (1974 to current), APA PsycInfo and Web of Science databases.

The preliminary search strategy (e.g., MEDLINE (Ovid)) will be adapted for use in other electronic bibliographic databases. Additional searches will be conducted in ClinicalTrials.gov ( www.ClinicalTrials.gov), the World Health Organization International Clinical Trials Registry Platform (ICTRP) and search Portal (apps.who.int/trialsearch/). The results of the literature search using the search terms listed above will be then checked for duplications.

We will check reference lists in included studies and any relevant systematic reviews identified. We will also contact researchers and organizations in the field to identify ongoing and unpublished trials if we identified them in clinical trial registers such as, the ClinicalTrials.gov, the International Clinical Trials Registry Platform (ICTRP) or the German Clinical Trials Register (DRKS); or if we miss any data in the found RCT. To identify as much relevant evidence as possible, we will also search relevant grey literature sources such as reports, dissertations theses and conference abstracts.

### Search strategy

The search will include the study type according to the PICO(S) scheme. As such, no additional filters will be integrated.

The review is planned as a living review. The search update is planned to be done twice a year (April and October). If more than three articles for one of the primary outcomes will be identified, the meta-analysis will be repeated respectively, and the new updated results will be published. Within the review team there are at least three researchers with a research team and a lifetime position (Y.N., U.M., B.W., A.L.) who will be responsible for this.

### Study records

***Overall data management***

To standardize the process of data collection and analysis and ensure the overall quality, we will proceed as follows.

1. We will separate the process into different tasks and set up a team for each task. The team will be guided by the first and second author to ensure the quality and standardization of the task.
2. For some tasks (how to use Rayyan and Redcap), we will develop a short training program for the teams. The training will be supported by manuals that refer to each step of the process.
3. The whole group has regular meetings to discuss difficulties and ensure standardization. All decisions and changes will be recorded.

### Study selection process

After removing duplicates both manually and electronically, at least two review authors of the whole author team will independently screen the titles and abstracts of the citations retrieved by the searches for relevance using the Rayyan software ( Ouzzani *et al.*, 2016; https://www.rayyan.ai/) Duplicates will be removed with help from the software. After this initial assessment, we will obtain full-text copies of all potentially relevant studies. Again at least two review authors will independently check the full papers for eligibility, resolving any disagreements by consensus and the input of a third review author. We will contact the corresponding authors, if studies cannot be found via the data sources or librarians.

Afterwards we will record the reasons for exclusion of studies obtained as full text in the excluded studies' table. Where there are several reports of a study, we attempt to obtain all reports. The process of the study selection will be presented in a PRISMA flowchart ( Page *et al.*, 2021).

### Data collection process

The same review authors responsible for the study selection will independently perform data extraction using a standardized data extraction form. All authors will be involved in data extraction.

We will pilot the data extraction form using a representative sample of studies to identify missing items or unclear coding instructions. Any disagreements will be resolved by consensus or by third-party adjudication. We will enter data into the Redcap software ( https://projectredcap.org/software/) (and check for accuracy and consistency against the data extraction form. The review authors will not be blinded to the authors and sources.

The final number of studies will equally be shared within the whole author team.

### Data items

We will record the following information in a 'Characteristics of included studies' table by using Redcap.

- General information: first author’s name; date of data extraction; study ID; author’s contact address (if available); citation of paper; and trial objectives.
- Detailed description of included participants (number or % of male/female, BMI, height, weight, comorbidities, MoCA/MMSE score, accommodation [independent, nursing home etc.] as well as socioeconomic level.
- Trial details: trial design; location; setting; sample size; inclusion and exclusion criteria; comparability of groups; length of follow-up; stratification reported in the study methods; stopping rules; and funding sources.
- 'Risk of bias' assessment: sequence generation; allocation concealment; blinding (participants, personnel, outcome assessors); incomplete outcome data; selective outcome reporting; and other biases (recall bias).
- Characteristics of participants: age; gender; ethnicity; health conditions; level of mobility/activity (according to SPPB, TUG, or gait speed); fitness level at baseline (according to IPAQ, GPAQ, or physical measurements like VO _2_max ( Stelmach, 2018), falls in the last 6 or 12 months before the intervention; Fear of Falling; Level of Global Cognition (assessed with the MMSE or MoCA); years of education; number of randomized participants; analyzed; lost to follow-up; and dropouts in each arm (with reasons).
- Interventions: experimental and control interventions; timing of intervention; intensity, duration and frequency of intervention/s; adherence (compliance) with experimental and control interventions and associated data; who delivered the intervention; and additional co-interventions (such as motivational strategies).

### Outcomes and prioritization

The primary outcomes will include parameters representing functional mobility: Short Physical Performance Battery (SPPB); Timed up and Go test (TUG), 6minute walk test 6MWT, and walking capacity: Walking distance and number of steps per day (according to accelerometer data). The secondary outcomes will include additional gait parameters (cf. Table 1)

***Outcomes measures:***

We plan the following analyzing steps:

1 Effects of the intervention vs (a) the active or (b) inactive control groups
2 Effects with respect to the frequency and duration (time (i.e. weeks or months), volume (total duration in minutes))
3 We will also consider comparisons of high- versus low-intensity interventions
4 If possible, we will also compare interventions from the different categories (cardiovascular exercise, resistance exercise, motor-coordinative exercise, multicomponent exercise, mind-body exercise, exergames, hybrid exercise, and concurrent training) as a network analysis
5 Influence of the moderator variables described in the "subgroup analysis" section

The final proofing of the data extraction will be done by the first and second author of the study. We will collect as much information as we can on control interventions, including assessing what 'usual care' comprises. We will retrieve data from both full-text and abstract reports of studies, including those with multiple reports. Where information is insufficient, we will contact the study authors for additional details.

### Risk of bias in individual studies

The whole author team subdivided into pairs including experienced review authors will independently assess the risk of bias using Cochrane's Risk of Bias 2 tool ( Sterne *et al.*, 2019). Any disagreement will be resolved by consensus or third-party adjudication. We will rate the risk of bias as either low, some concerns, or high by the use of the Risk of Bias 2 tool (ROB2) based on the following domains:

- bias arising from the randomization process (the allocation sequence was random; the allocation sequence was adequately concealed; baseline differences between intervention groups suggesting a problem with the randomization process).
- bias due to deviations from intended interventions (participants were aware of their assigned intervention during the trial; people delivering the interventions were aware of participants’ assigned intervention during the trial, deviations from the intended intervention arose because of the experimental context, an appropriate analysis was used to estimate the effect of assignment to intervention, important non-protocol interventions were balanced across intervention groups; (if applicable) failures in implementing the intervention could have affected the outcome; (if applicable) study participants adhered to the assigned intervention regimen; (if applicable) an appropriate analysis was used to estimate the effect of adhering to the intervention.
- bias due to missing outcome data (data for this outcome were available for all, or nearly all, participants randomized; (if applicable) there was evidence that the result was not biased by missing outcome data; (if applicable) missingness in the outcome was likely to depend on its true value (e.g., the proportions of missing outcome data, or reasons for missing outcome data, differ between intervention groups).
- bias in measurement of the outcome (data for this outcome were available for all, or nearly all, participants randomized; (if applicable) there was evidence that the result was not biased by missing outcome data; (if applicable) missingness in the outcome was likely to depend on its true value (e.g., the proportions of missing outcome data, or reasons for missing outcome data, differ between intervention groups).
- bias in selection of the reported result (data for this outcome were available for all, or nearly all, participants randomized; (if applicable) there was evidence that the result was not biased by missing outcome data; (if applicable) missingness in the outcome was likely to depend on its true value (e.g., the proportions of missing outcome data, or reasons for missing outcome data, differ between intervention groups).

We will contact study authors for additional information about the studies included, or for clarification of the study methods if required.

The domain with the highest risk of bias per outcome will be used to report an overall risk of bias for each respective outcome. Only studies with a low to medium risk of bias will be included in the analyses. Studies exhibiting a high risk of bias will be reported but not included in meta-analyses. Results of the risk of bias assessment will be made available on reasonable request.

We will provide the risk of bias assessment results in the review through standard tables. The assessed risk of bias will be visualized in dedicated figures and, if feasible, in visualizations of meta-analyses as well as tables such as the summary of findings and GRADE ( GRADEpro GDT).

### Measures of treatment effect

We will process data in accordance with the *Cochrane Handbook for Systematic Reviews of Interventions* ( Deeks *et al.*, 2019; Higgins *et al.*, 2019).

All described functional mobility outcomes are continuous measures. For these continuous outcomes, we will present the mean difference (MD) with 95% CIs where the same outcome measure was used. If more than one study measures the same outcome using different tools, we will calculate the standardized mean difference (SMD) and 95% CI using the inverse variance method in R or Stata. This method enables pooling of the adjusted and unadjusted treatment effect estimates reported in the individual studies or calculated from data presented in the published article.

For any dichotomous outcomes (with respect to potential subgroup analysis) resulting risk ratios (RR) will be calculated.

For cluster RCTs data adjustments will be done according to the *Cochrane Handbook for Systematic Reviews of Interventions*. Ideal information to extract will be a direct estimate of the required effect measure and standard error from analysis made by the authors. Alternatively, data will be analysed as if each cluster was a single individual, using a summary measurement from each cluster.

### Unit of analysis issues

This review of RCTs will include studies with two or more parallel groups, cross-over and cluster designs.

For trials with multiple arms, we will include multiple pair-wise comparisons (intervention versus control) in analyses, but to avoid the same group of participants being included twice, we will 'split' the control group by distributing the number of control group participants to each analysis considering whether (1) no irreversible events such as mortality had occurred, and (2) appropriate statistical approaches had been used.

For the crossover randomized trials, the unique design of the trials will be considered with respect to the multiple treatments or interventions in a predetermined sequence. Within the review, the unit of analysis issues addressed in individual studies will be reported with giving details about the statistical methods used, the handling of carryover and period effects, and any assumptions made. To account for carryover effects in the analysis, the data from the different periods will be treated separately. Data from the first period in crossover trials will be included. Within crossover randomized trials, washout periods durations for physical training intervention effects in older adults are not precisely known but are believed to be lasting months or even years. Due to the inclusion of an aging population, this would lead to important changes and possibly mortality between periods. Therefore, including following periods do not seem suitable for this meta-analysis and only Data from the first period in crossover trials will be included.

For data from cluster trials, we will consider whether the reported data analysis had appropriately considered the aggregated nature of the data. When a direct estimate of the required effect measure from an analysis that properly accounts for the cluster design is not available, to avoid unit-of-analysis error, data will be analyzed as if each cluster was a single individual, using a summary measurement from each cluster.

Approximately analyses will be performed in case of randomization performed on the individuals rather than the clusters in the trial, and if if the following information can be extracted: the number of clusters randomized to each intervention group, the total number of participants in the study or the average (mean) size of each cluster, the outcome data ignoring the cluster design for the total number of individuals and an estimate of the intracluster (or intraclass) correlation coefficient (ICC). The design effect will be calculated as 1 + (m - 1) * ICC, where "m" is the average cluster size to adjust the sample size calculation. Moreover, sensitivity analyses to assess the impact of different ICC values or clustering assumptions on the results to evaluate the robustness of the findings will be conducted.

Moreover, sensitivity analyses to assess the impact of different ICC values or clustering assumptions on the results to evaluate the robustness of the findings will be conducted.

### Dealing with missing data

We will contact the corresponding authors of the included studies for any key missing numerical data or information on their trial. If data cannot be retrieved, we will conduct sensitivity analysis to explore the effects of missing data (incomplete outcome data) on the treatment effect.

If a study does not report SDs for continuous outcomes, we will calculate these from standard errors, CIs, or exact probability (P) values where possible. We will not impute missing SDs.

### Assessment of heterogeneity

The decision about whether to combine the results of individual studies depends on an assessment of clinical and methodological heterogeneity. To investigate heterogeneity among the included studies, various potential sources of variation are explored. These factors include differences in participant characteristics (see inclusion criteria), effect of the type of exercise intervention, exercise intensity and outcome measures. The investigation of heterogeneity may suggest that differences in baseline levels of functional mobility and comorbidity profiles across studies will contribute to heterogeneity in effect sizes.

If we consider studies sufficiently homogeneous in their study design (participants, interventions, measurement tools), we will carry out meta-analyses and assess the statistical heterogeneity. The heterogeneity-rating will be displayed in the results section. We will assess statistical heterogeneity of treatment effects between trials by funnel plots, Chi² test with a significance level at P < 0.10, and the I² statistic. We will base our interpretation of the I² values suggested by Higgins, Li, & Deeks (2019):

- 0% to 40% might not be important;
- 30% to 60% may represent moderate heterogeneity;
- 50% to 90% may represent substantial heterogeneity; and
- 75% to 100% may represent very substantial ('considerable') heterogeneity.

Cases of considerable heterogeneity will be reported, further investigated by subgroup analyses, and considered interpreting the results of affected meta-analyses.

### Assessment of reporting biases

We will assess reporting bias according to ROB 2 and do additional quality checks based on the characteristics of the included studies (e.g., if only studies with small sample sizes indicate positive findings), and if information obtained from contacting experts and authors of studies suggests there are relevant unpublished studies. In the NMA, a comparison-adjusted funnel plot will be utilised to identify potential small-study effects ( Chaimani & Salanti, 2012)

Afterwards, to explore the possibility of publication and other reporting biases, we will conduct tests for funnel plot asymmetry for analyses that contain more than 10 data points. (according to Higgins *et al.*, 2019). This will be done for each comparison of the network if it is feasible. Moreover, comparison-adjusted funnel plots will be conducted to identify possible small-study effects in the network meta-analysis. It is unlikely that one of the authors on this review will be an author of one of the reviewed articles. If this happens, these authors will not be involved in the risk of bias assessment for their own study or be involved in the GRADE assessment that uses data from their own study.

### Data synthesis

When considered appropriate, we will pool the results of comparable studies using random-effects models. The choice of model will be guided by careful consideration of the extent of heterogeneity and whether it can be explained, in addition to other factors, such as the number and size of included studies. We will use 95% CIs throughout ( Cumming, 2014)

When considered appropriate, we will pool data using the generic inverse variance method in R or Stata. This method enables pooling of the adjusted and unadjusted treatment effect estimates (rate ratios or risk ratios) reported in the individual studies or calculations from data presented in the published article (see Measures of treatment effect). We will consider not pooling data where there is considerable heterogeneity (I ^2^ ≥ 75%) that cannot be explained by the diversity of methodological or clinical features among trials. Where pooling data is inappropriate, we will still present trial data in the tables, for illustrative purposes, and in the text.

We will use a random-effect model, considering the correlated treatment effects in multi-arm studies. We assume a common estimate for heterogeneity variance across different comparisons.

To evaluate the extent to which the treatments are connected, we will provide a network plot of our outcomes. For each comparison, we will report the estimated treatment effect along with its 95% CI. We will graphically present the results using forest plots, with comparisons of passive and active control groups as reference treatments.

### Meta-bias(es)

With additional mixed treatment network meta-analysis (NMA), we aim to compare multiple treatments in the context of meta-analysis. It incorporates direct comparisons of interventions within randomized controlled trials and indirect comparisons across trials based on a common comparator. Through NMA, we will estimate the relative effectiveness of multiple interventions, even if they haven't been directly compared in primary studies.

The analysis will start with a standard pairwise meta-analysis of the intervention effects of the different identified exercise interventions to understand the efficacy of individual interventions. At this stage a mixed treatment comparison is planned to combine evidence from both direct and indirect comparisons to estimate relative treatment effects (consistancy model). If possible, transitivity will be evaluated by comparing the distribution of effect modifiers across the different comparisons for studies with the same study characteristics and aims. A network plot will be created to visualize the available direct connections between the treatments. In addition to examining the overall effects of exercise interventions, the NMA is used to explore the influence of various study levels or participant-level characteristics (e.g., age, gender, exercise intensity) as moderators or subgroup analyses. Depending on potential identified inconsistency, adjustments to the analysis will be undertaken (e.g., incorporating study level covariates).

Meta-regressions will examine the relationship between one or more moderators and the treatment effect. The treatment effect will be the dependent variable and the selected moderators will be independent variables. The estimation of the coefficients (β values) for each moderator will be reported for the strength and direction of their association with the treatment effect. The examined p-values associated with each moderator in the meta-regression model indicate if the moderator has a statistically significant effect on the treatment effect. Additionally, we will conduct confidence intervals for the moderator coefficients to assess the precision of the estimated effects.

It is planned to use the R or Stata to conduct the network meta-analyses and mixed treatment comparisons.

Where outcome data from individual trials are not sufficiently similar to be combined within meta-analyses, or for studies excluded from meta-analysis due to low quality (according to RoB2) we will describe results from clinically comparable trials narratively using the synthesis without meta-analysis (SWiM) approach ( Campbell *et al.*, 2020). We will report information by grouping studies (e.g., by population, outcome, study design), and describing the standardised metrics and any transformations used, synthesis methods, the criteria used to prioritize results for synthesis (e.g. based on study design, risk of bias assessment), and limitation of the synthesis.

### Confidence in cumulative evidence

Moderator analysis will explore the impact of categorical and metric variables on the study effect sizes. With categorical variables, we will analyze whether different levels of a factor (e.g., intervention type) might explain variability in results. Metric variables in moderator analysis (meta-regression) allow the examination of the influence of continuous variables, like dosage or duration, on effect sizes.

We plan to carry out the following moderator analyses when sufficient data exist.

Related to participants:

- Sex: male, female.
- Age: 60–70, 71–80, >80
- Level of mobility/activity at baseline: SPPB </> 8; TUG </>15 sec; gait speed < 0.8 m/s; .08-1.0 m/s; > 1.0 m/s
- Health conditions: no disease, orthopedic diseases, brain diseases, mental disorders, other diseases
- Falls in the last 6 or 12 months before the intervention: yes, no
- Fear or concerns of falling (reported or rated by the FES-I): low, moderate, high concern about falling; reported or rated by the FES-I
- Level of global cognition: MMSE, MOCA at baseline (< 24 or ≥ 24)
- Years of education: <8,8-10, >10 years

Fitness level at baseline: low, moderate or high according to IPAQ or GPAQ or physical measurements like VO _2_max ( Stelmach, 2018)

Related to the interventions:

- The frequency of the exercise training (number of sessions a week)
- The intensity of the prescribed physical activity
- The type of physical exercise intervention
- The time (duration) of the intervention (number of weeks)
- The time (duration) in average of each session (in minutes)
- Related to the study design: Type of control group (active vs. non active; type of intervention)

### Sensitivity analysis

Where possible, we will assess the robustness of our findings by conducting sensitivity analyses.

We will examine the effects of the following aspects:

- Inclusion of trials at high or unclear risk of selection bias due to inadequate concealment of allocation.
- Inclusion of trials at high or unclear risk of detection bias due to inadequate blinding of outcome assessors.
- Inclusion of trials at high or unclear risk of attrition bias due to incomplete outcome data.
- The effect of time on the impact of the intervention (i.e. comparing differences in treatment effect over time earlier trials versus later trials).
- The choice of statistical model for pooling (fixed-effect versus random-effects).

### Summary of findings and assessment of the certainty of the evidence

We will create summary of findings (SoF) tables for all our main comparisons of the NMA ( Yepes-Nuñez *et al.*, 2019). The summary of findings tables will report the comparisons findings of: exercise interventions versus active or inactive controls; exercise type and potential effects on functional mobility. The main comparatives will be the following outcomes (separate table per comparison)

- The Short Physical Performance Battery (SPPB)
- Timed up and Go test (TUG)
- 6MWT
- Walking distance/ number of steps per day
- Gait performance

The summary of finding tables will include: a description of patient populations included in the comparison, the intervention, outcome and setting/settings. The tables will also include total participant number, relative effect for the outcomes of interest [reported as odds ratio, credible intervals], anticipated absolute effect, certainty of evidence [according to GRADE], ranking of treatment [using SUCRA ranking and credible intervals], and interpretation (eg., positive or negative direction) of findings. The tables will also include a graphical presentation of the comparisons ( Brignardello-Petersen *et al.*, 2018; Mbuagbaw *et al.*, 2017; Salanti *et al.*, 2011). We will use the GRADE approach to assess the quality of evidence as it relates to the primary and secondary outcomes listed in the Types of outcome measures section. In assessing the quality of evidence using the GRADE approach, we will specify the comparisons and timing of follow-up to be included in the GRADE assessment and SoF table(s) ( Brożek *et al.*, 2011; Schünemann *et al.*, 2022). Additionally, we will explicitly state whether adverse effects of the interventions will be included in the SoF and GRADE assessments Within the GRADE the quality rating ‘high’ is reserved for a body of evidence based on randomised controlled trials. We may downgrade the quality rating to 'moderate', 'low’, or 'very low' depending on the presence and extent of five factors: study limitations, inconsistency of effect, imprecision, indirectness, and publication bias. Two people will work independently to assess the certainty of the body of evidence and third will resolve disagreement.

## Discussion

A growing number of older adults worldwide are losing their independence due to decreased functional mobility ( Braun *et al.*, 2022). Balance and walking are essential for independence as they are key factors of locomotion. The positive effect of physical activity on mobility has been well documented ( Taylor *et al.*, 2023; Valenzuela *et al.*, 2023). However, a sedentary lifestyle ( Harvey *et al.*, 2015) as well as huge variability in functional mobility ( Netz *et al.*, 2019) is common in older adults. Not all exercise regimens are universally effective, and inter-individual differences in response to exercise exist ( Bonafiglia *et al.*, 2021). Moreover, adaptations to physical exercises are specific to the training contents (e.g., balance or walking capacity). Therefore, there is an urgent need for assessing which exercise programs and delivery modes are effective to improve older adult’s functional mobility.

Surprisingly, to date, no systematic review has been published which systematically assessed aspects of individualization as well as the frequency, intensity, type, and time (duration) (F.I.T.T.) of the exercises, to derive specific dose-response relationships with respect to the exercise effects on functional mobility. A recent review regarding training to increase mobility and functioning in older adults included independent community-dwelling frail older adults but excluded other populations such as non-frail older adults or those living in residential homes ( Treacy *et al.*, 2022). Moreover, the review disregarded training principles concerning F.I.T.T. principles. Other reviews focused on certain training interventions like progressive resistance training (PRT) for improving physical functions in older adults but excluded other types of exercise such as endurance and balance training ( Liu & Latham, 2009). Other reviews focused on outcomes that could be associated with mobility impairment like falls ( Treacy *et al.*, 2022), or fear of falling ( Kumar *et al.*, 2016), but did not assess functional mobility. Numerous other reviews examined multiple exercise regimens, but they all focused on specific populations, e.g. frail older adults ( Treacy *et al.*, 2022), adults post-hip fracture surgery ( Fairhall *et al.*, 2022), acutely hospitalized older adults ( Hartley *et al.*, 2022), patients with hip or knee osteoarthritis ( Regnaux *et al.*, 2015) or ankylosing spondylitis ( Regnaux *et al.*, 2019), patients with multiple sclerosis ( Rietberg *et al.*, 2005), patients with cerebral palsy ( Ryan *et al.*, 2017) and other similar specific population subgroups.

Our intention in this comprehensive living review and meta-analysis is to identify the optimal exercise characteristics to increase functional mobility (based on objective, well-established measures) in older adults. We will utilise an evidence-based approach. Subsequently, policymakers, health professionals, and practitioners will be able to provide dedicated and effective evidence-based exercise programs for improving functional mobility in older adults for both, prevention (in the healthy population), as well as for rehabilitation (in the mobility-impaired population) and consequently, promote the independence of older adults.

The proposed review has the potential to provide evidence-based data on exercise interventions that target functional mobility.

In summary, the planned review goes beyond the already published work as it aims to analyze a range of functional mobility outcomes (walking capacity, gait, balance, and overall physical activity levels) concerning several moderators including:

•   physical and health status

•   exercise interventions with respect to F.I.T.T principles

Furthermore, the evidence of the studies included will be reported according to GRADE guidelines.

The GRADE approach for quality of evidence assessment will be utilised for all outcomes and measures, including all factors referred to by these guidelines ( Brignardello-Petersen *et al.*, 2018).

## Ethics and consent

Ethical approval and consent were not required

## Data Availability

No data are associated with this article. Open science framework (OSF): Evidence-based exercise recommendations to improve functional mobility in older adults - A study protocol for living systematic review and meta-analysis _material https://doi.org/10.17605/OSF.IO/4V5P3 ( Wollesen, 2024) This project contains the following extended data: Preliminary Search Strategy.docx PRISMA-P_checklist.docx Data are available under the terms of the CC0 1.0 Universal license. The PRISMA-P checklist for this review can be found here: https://doi.org/10.17605/OSF.IO/4V5P3 ( Wollesen, 2024) Data are available under the terms of the CC0 1.0 Universal license.
