# Supplementary material for: Evidence-based exercise recommendations to improve functional mobility in older adults - A study protocol for living systematic review and meta-analysis
Source: Open Res Eur. 2025 Jan 14;4:202. Originally published 2024 Sep 11. [Version 2] doi: 10.12688/openreseurope.17823.2 (PMC11770255; doi:10.12688/openreseurope.17823.2)
Supplement: Peer review history

# Reviewer report: Reviewer response for version 2

**DOI:** 10.21956/openreseurope.20770.r49731

**Published:** 2025-01-27

**Authors:** Paulo Cesar Barauce Bento, Neiry Arsie

**Affiliations:** 1 Department of Physical Education, Graduate Program in Physical Education, Federal University of Paraná, Curitiba, Brazil; 2 Phisycal Education, Federal University of Paraná, Curitiba, PR, Brazil

Once again, I appreciate the opportunity to review the manuscript. I believe the authors have fully addressed the requests and clarifications asked.

Is the study design appropriate for the research question?

Yes

Is the rationale for, and objectives of, the study clearly described?

Yes

Are sufficient details of the methods provided to allow replication by others?

Yes

Are the datasets clearly presented in a useable and accessible format?

Not applicable

Reviewer Expertise:

My area of expertise is the effects of physical activity and exercise on physical function in older adults.

We confirm that we have read this submission and believe that we have an appropriate level of expertise to confirm that it is of an acceptable scientific standard.

# Reviewer report: Reviewer response for version 2

**DOI:** 10.21956/openreseurope.20770.r49730

**Published:** 2025-01-15

**Authors:** Luis A Berlanga

**Affiliations:** 1 Universidad Francisco de Vitoria, Pozuelo de Alarcón, Community of Madrid, Spain; 2 Centro de Estudios Universitarios Cardenal Spínola CEU, Sevilla, Spain

Authors have conducted a careful review of the comments provided by the reviewers and have made the appropriate modifications. No further modifications to the manuscript are necessary from my point of view. I appreciate the work of all co-authors and the relevance of the article.

Is the study design appropriate for the research question?

Yes

Is the rationale for, and objectives of, the study clearly described?

Yes

Are sufficient details of the methods provided to allow replication by others?

Yes

Are the datasets clearly presented in a useable and accessible format?

Not applicable

Reviewer Expertise:

Exercise Physiology; Physical Fitness Assessment

I confirm that I have read this submission and believe that I have an appropriate level of expertise to confirm that it is of an acceptable scientific standard.

# Reviewer report: Reviewer response for version 1

**DOI:** 10.21956/openreseurope.19267.r44981

**Published:** 2024-11-06

**Authors:** Luis A Berlanga

**Affiliations:** 1 Universidad Francisco de Vitoria, Pozuelo de Alarcón, Community of Madrid, Spain; 2 Centro de Estudios Universitarios Cardenal Spínola CEU, Sevilla, Spain

Authors described a very well-designed and rigorous study protocol to perform a systematic review with meta-analysis for helping policymakers, health professionals, and practitioners to achieve practical tools and knowledge for developing effective evidence-based exercise programs for improving functional mobility in older adults.

 The introduction presents the content in a clear, orderly and rigorous manner; state of the art is well reflected. The authors correctly define the concept of ‘functional mobility’. However, in my opinion, it would be more accurate to refer to this concept as ‘functional capacity’, as this term is much more commonly used in the scientific literature to describe what authors described. Readers may confuse ‘functional mobility’ with the concept of ‘mobility’, associated with flexibility or range of motion; therefore, in case authors refused to replace the term, I would suggest that it should be used throughout the manuscript as ‘functional mobility’ to avoid possible confusion (i.e.: first sentence in ‘Objective’ section; or the 3rd sentence in 'Discussion').

 I consider that the section ‘Short Physical Performance Battery (SPPB) and Timed Up and Go (TUG)’ should not be titled with the name of the tests, but with the outcomes that these tests assess (like the previous section, which is titled ‘Walking capacity and Gait performance’ instead of ‘6MWT’). A proposed title for this section could be ‘Other functional mobility assessments’, since the tests mentioned assess different components of functional capacity (strength, balance...), and are related to the ability to perform activities of daily living, risk of falls, etc.

 Regarding the type of training interventions, it would be important to mention the importance of resistance training focused on muscle power improvement due to its importance for the increase of functional capacity in older people (i.e.: 10.1093/gerona/glx216)-ref-2.

 The proposed methodological design is solid and rigorous. However, in the section ‘Types of participants’ it is not clear whether the age of inclusion is greater than 60 or 50 years old; please clarify. In addition, please delete the last sentence within ‘Types of intervention’ section as this information is repeated. Furthermore, please do not repeat the explanation of abbreviations already used within the manuscript (section ‘Primary outcomes’ and ‘Outcomes and prioritization’).

 In page 12, authors explained that fitness would be classified according to IPAQ. Nevertheless, this questionnaire does not assess fitness, but the amount of physical activity daily and sedentary behavior. Obviously, both are related to fitness, but IPAQ has not been designed to assess physical condition. It would be more accurate to classify the results according to cardiorespiratory fitness assessed with 6MWT, for example, using different values (meters covered within the 6-min test) for each sex (male, female) and each range of age (60-70, 71-80, and >80) (author may be interested in this publication: 10.1183/09031936.00194909). On the other hand, results from IPAQ could be used to classify participants as low, moderate or high physically active; which it differs from fitness level.

 Additionally, I consider that training volume should be also important to be registered (numbers of set, repetitions and exercise per training session); and not only duration of each session since time may not reflected the amount of work performed. This concern it would be important even though American Collège of Sport Medicine commonly uses FITT principles, where training volume may not be clearly described.

Is the study design appropriate for the research question?

Yes

Is the rationale for, and objectives of, the study clearly described?

Yes

Are sufficient details of the methods provided to allow replication by others?

Yes

Are the datasets clearly presented in a useable and accessible format?

Not applicable

Reviewer Expertise:

Exercise Physiology; Physical Fitness Assessment

I confirm that I have read this submission and believe that I have an appropriate level of expertise to confirm that it is of an acceptable scientific standard, however I have significant reservations, as outlined above.

## References

[1] : The 6-min walk distance in healthy subjects: reference standards from seven countries. *Eur Respir J* .2011;37(1) : 10.1183/09031936.00194909 150-6 10.1183/09031936.00194909 20525717

[2] : Skeletal Muscle Power Measurement in Older People: A Systematic Review of Testing Protocols and Adverse Events. *J Gerontol A Biol Sci Med Sci* .2018;73(7) : 10.1093/gerona/glx216 914-924 10.1093/gerona/glx216 29309534

# Response

**Published:** 2024-12-13

**Authors:** Bettina WOLLESEN

**Response to reviewer 2**   Authors described a very well-designed and rigorous study protocol to perform a systematic review with meta-analysis for helping policymakers, health professionals, and practitioners to achieve practical tools and knowledge for developing effective evidence-based exercise programs for improving functional mobility in older adults. **Answer: **We thank you for your overall estimation and the time you spent in critically reviewing our manuscript. We included your suggestions into the manuscript asnd highlighted them in light blue.

 The introduction presents the content in a clear, orderly and rigorous manner; state of the art is well reflected. The authors correctly define the concept of ‘functional mobility’. However, in my opinion, it would be more accurate to refer to this concept as ‘functional capacity’, as this term is much more commonly used in the scientific literature to describe what authors described. Readers may confuse ‘functional mobility’ with the concept of ‘mobility’, associated with flexibility or range of motion; therefore, in case authors refused to replace the term, I would suggest that it should be used throughout the manuscript as ‘functional mobility’ to avoid possible confusion (i.e.: first sentence in ‘Objective’ section; or the 3rd sentence in 'Discussion').  

**Answer: **We thank the reviewer for this suggestion and changed the whole manuscript accordingly.

 I consider that the section ‘Short Physical Performance Battery (SPPB) and Timed Up and Go (TUG)’ should not be titled with the name of the tests, but with the outcomes that these tests assess (like the previous section, which is titled ‘Walking capacity and Gait performance’ instead of ‘6MWT’). A proposed title for this section could be ‘Other functional mobility assessments’, since the tests mentioned assess different components of functional capacity (strength, balance...), and are related to the ability to perform activities of daily living, risk of falls, etc.  

**Answer:** We agree with the reviewers idea and changed it accordingly.

 Regarding the type of training interventions, it would be important to mention the importance of resistance training focused on muscle power improvement due to its importance for the increase of functional capacity in older people (i.e.: 10.1093/gerona/glx216)-ref-2.  

**Answer:** We thank the reviewer for this advice and inserted this aspect on page 5 as follows:   “Resistance training helps to increase muscle strength in the lower limb, which increases gait stability (Brach & Van Swearingen, 2013; Fragala et al., 2019) and muscle power improvement increases functional capacity (Alcazar et al., 2018).”

 The proposed methodological design is solid and rigorous. However, in the section ‘Types of participants’ it is not clear whether the age of inclusion is greater than 60 or 50 years old; please clarify.  

**Answer: **We agree with the reviewers conconcern and changed the section as follows: Types of participants We will include studies involving participants aged > 50, according to the WHO definition of "older adults" (mean age of 60 years ± SD, with a minimum age of 50).   And in the next section:   Firstly, we will meticulously assess each study's eligibility criteria to ascertain if they meet our age inclusion (mean age of 60 years, age range starting at 50 years) and exclusion criteria for specific conditions.    

 In addition, please delete the last sentence within ‘Types of intervention’ section as this information is repeated.   Answer: We thank the reviwer for this advice and deleted the sentence as suggested.   Furthermore, please do not repeat the explanation of abbreviations already used within the manuscript (section ‘Primary outcomes’ and ‘Outcomes and prioritization’).

**Answer: **We changed the text according to this advice.

 In page 12, authors explained that fitness would be classified according to IPAQ. Nevertheless, this questionnaire does not assess fitness, but the amount of physical activity daily and sedentary behavior. Obviously, both are related to fitness, but IPAQ has not been designed to assess physical condition. It would be more accurate to classify the results according to cardiorespiratory fitness assessed with 6MWT, for example, using different values (meters covered within the 6-min test) for each sex (male, female) and each range of age (60-70, 71-80, and >80) (author may be interested in this publication: 10.1183/09031936.00194909). On the other hand, results from IPAQ could be used to classify participants as low, moderate or high physically active; which it differs from fitness level.  

**Answer:** We thank the reviwer for this comment. We changed this section as follows:   Characteristics of participants: age; gender; ethnicity; health conditions; level of mobility/activity (according to SPPB, TUG, or gait speed); physical activity level at baseline (according to IPAQ, GPAQ, fitness level at baseline with measurements like VO _2_max (Stelmach, 2018) or walking capacity using the 6MWT, falls in the last 6 or 12 months before the intervention

 Additionally, I consider that training volume should be also important to be registered (numbers of set, repetitions and exercise per training session); and not only duration of each session since time may not reflected the amount of work performed. This concern it would be important even though American Collège of Sport Medicine commonly uses FITT principles, where training volume may not be clearly described.  

**Answer:** We definitely agree with this comment! We added the training volume into the manuscript. We classified this in our internal papers as “training characteristics” but did not transfer this into the study protocol. We now added the volume into the protocol as follows:   Interventions: experimental and control interventions; timing of intervention; intensity, duration, volume and frequency of intervention/s;

# Reviewer report: Reviewer response for version 1

**DOI:** 10.21956/openreseurope.19267.r44568

**Published:** 2024-10-28

**Authors:** Paulo Cesar Barauce Bento, Neiry Arsie

**Affiliations:** 1 Department of Physical Education, Graduate Program in Physical Education, Federal University of Paraná, Curitiba, Brazil; 2 Phisycal Education, Federal University of Paraná, Curitiba, PR, Brazil

Firstly, I would like to thank you for the opportunity to review the article “Evidence-based exercise recommendations to improve functional mobility in older adults—A study protocol for living systematic review and meta-analysis.”

 The proposed article develops a comprehensive systematic review and meta-analysis research regarding evidence-based exercise recommendations for functional mobility in older adults. Given the various existing protocols on this topic and the difficulty of standardization, I consider this idea extremely important.

 The article is well written, presenting an introduction with a solid literature review and adequately explaining the study’s rationale and objectives. Given the various existing protocols on this topic and the difficulty of establishing a dose-response relationship of physical exercise for mobility improvement, I consider this idea extremely important.

 The methodological aspects are presented in detail, but some aspects deserve attention:

- I missed a definition of F.I.I.T. It is in the abstract or right at the introduction but only appears at the end of the discussion.
- The search mechanisms are clear, but the feasibility of searching for unpublished data, depending on the availability of the authors, is confusing. It also mentions different languages, which I consider quite broad.
- The keywords are not listed in the MeSH terms.
- The study selection initially indicates that articles evaluating people aged 50 and over will be included, but the study is for the aged population. At another point in the methods, it appears that people aged 60 and over will be included. Please clarify this point.
- Regarding the outcome of “functional mobility,” it was unclear why the daily walking distance and the number of daily steps were included among the primary outcomes. These outcomes are more directly related to the level of physical activity and could be on the list of secondary outcomes or just included as moderator variables.
- The description of the analyses for the meta-analysis is presented in detail, as are the strategies for presenting the results.

Is the study design appropriate for the research question?

Yes

Is the rationale for, and objectives of, the study clearly described?

Yes

Are sufficient details of the methods provided to allow replication by others?

Yes

Are the datasets clearly presented in a useable and accessible format?

Not applicable

Reviewer Expertise:

My area of expertise is the effects of physical activity and exercise on physical function in older adults.

We confirm that we have read this submission and believe that we have an appropriate level of expertise to confirm that it is of an acceptable scientific standard, however we have significant reservations, as outlined above.

# Response

**Published:** 2024-12-13

**Authors:** Bettina WOLLESEN

Dear reviewers, we thank you for the time and effort you spent on reading and commenting our manuscript.  We revised the manuscript following your recommendations, highlighting the changes according to reviewer 1 in yellow and to reviewer 2 in light blue.    

 Firstly, I would like to thank you for the opportunity to review the article “Evidence-based exercise recommendations to improve functional mobility in older adults—A study protocol for living systematic review and meta-analysis.”

 The proposed article develops a comprehensive systematic review and meta-analysis research regarding evidence-based exercise recommendations for functional mobility in older adults. Given the various existing protocols on this topic and the difficulty of standardization, I consider this idea extremely important.

 The article is well written, presenting an introduction with a solid literature review and adequately explaining the study’s rationale and objectives. Given the various existing protocols on this topic and the difficulty of establishing a dose-response relationship of physical exercise for mobility improvement, I consider this idea extremely important.  

**Answer:** We thank the reviewer for this estimation.

 The methodological aspects are presented in detail, but some aspects deserve attention:

- I missed a definition of F.I.I.T. It is in the abstract or right at the introduction but only appears at the end of the discussion.

**Answer:** We agree with the reviewers advice and changed the sections as follows:   Abstract: *Background and objectives* This is a protocol for a living systematic review and meta-analysis. It will assess the effects of state-of-the-art exercise interventions designed to promote functional mobility. Therefore, after identifying all potential interventions, we will use the F.I.T.T. principles (frequency, intensity, time, type) as well as the physical and health status of the participants as moderators to analyse the mechanisms for the positive benefits of exercising. The main research questions are: Which exercise types are most beneficial for improving functional mobility in various populations of older adults? Which physical exercise characteristics in terms of frequency, intensity, time and duration will achieve the greatest benefit in terms of the defined outcomes, i.e, the functional mobility of older adults? Methods According to PRISMA guidelines will search databases like MEDLINE, APA Psych-Info and Web of Science. Inclusion criteria are: healthy older people ≥ 50 years, randomized-controlled trials including exercise intervention and a walking or mobility assessments (eg., TUG, SPPB) as an outcome measure. A preliminary search revealed more than 33,000 hits that will be screened by pairs of independent reviewers. The results will be summarized according to the effects regarding functional mobility and potential dose-response relations via respective meta-analysis.  

 Conclusion The systematic review will comprise the knowledge of the existing literature with regards to the effects of the physical activity interventions compared to an active or inactive control group. We will summarize the effects with respect to the F.I.T.T.. They provide a foundation for structuring an optimal exercise training program. If possible, we will also compare interventions from the different categories (eg. cardiovascular, resistance, motor-coordinative, multicomponent or mind-body exercise) as a network analysis and report the influence of moderator variables. Based on the results evidence-based guidelines following GRADE for physical exercise interventions to improve functional mobility in older adults will be provided.   Methods - **Types of interventions** **F.I.T.T. principle**   The F.I.T.T. principle, a widely-used framework in exercise science, is based on four key factors: frequency, intensity, time, and type. The F.I.T.T. principle provides a framework for the structure of exercise programmes, with a particular focus on the following four key elements: the frequency of exercise, the intensity of the workout, the duration of the exercise session, and the specific form of the activity. This principle is fundamental to the prescription of exercise, allowing for adjustments to be made to meet individual fitness levels and goals. As a result, it has become an enduring tool in the fields of health and fitness (Schmitz et al., 2010). The term 'frequency' pertains to the number of times an individual engages in physical activity on a weekly basis, typically expressed as the number of sessions completed per week. The evidence indicates that undertaking moderate-intensity aerobic exercise on three to five occasions per week is beneficial for cardiovascular health and overall wellbeing. Similarly, resistance training is recommended on two to three occasions per week to improve muscle mass and strength (Garber et al., 2011). The term 'intensity' refers to the level of effort or difficulty associated with a particular exercise. It is a crucial factor in the adaptation of exercise programmes to suit different fitness levels and goals. The intensity of an exercise can be gauged by a number of means, including heart rate, perceived exertion, or metabolic equivalents (METs). Higher intensities, such as those employed in high-intensity interval training (HIIT), have been demonstrated to confer distinct cardiovascular and metabolic benefits (Hwang et al., 2016). The term "time" refers to the duration of each exercise session. For example, the general recommendation is to engage in 150 minutes per week of moderate aerobic activity or 75 minutes of vigorous activity. The duration of the exercise session is adjusted based on the intensity. Shorter sessions can be effective if the intensity is higher, as demonstrated by Hillman et al. (2020) in the case of HIIT. The term "type" denotes the specific category of exercise, encompassing aerobic activities such as running and swimming, anaerobic exercises like weightlifting, and flexibility training. The different types of exercise have been shown to have varying beneficial effects on health. For example, aerobic exercise has been demonstrated to have positive effects on cardiovascular health, while resistance training has been shown to be effective in building muscle and bone strength (Lima et al., 2015).    

- The search mechanisms are clear, but the feasibility of searching for unpublished data, depending on the availability of the authors, is confusing. It also mentions different languages, which I consider quite broad.

**  Answer:  **We agree with the reviewers concerns and revised the section. We deleted the part incorporating different languages.   We clarified the second aspect as follws:   The search will span all eligible studies in English language accessible through databases (Lefebvre 2022). Only studies reporting trial results will be incorporated. In cases where multiple studies cover the same or overlapping populations, priority will be given to the study with the highest participant count or the one following pre-registration in a trial register. Reference lists from included studies and relevant systematic reviews will be examined. Additionally, efforts will be made to identify trials by checking clinical trial registers such as ClinicalTrials.gov, the International Clinical Trials Registry Platform (ICTRP), or the German Clinical Trials Register (DRKS). Any missing data from RCTs will also prompt further investigation.  

- The keywords are not listed in the MeSH terms.

**  Answer:** We thank you for this comment. Our original key words were: functional mobility, older adults,  physical training interventions, evidence-based exercise The MeSH terms adjusted keywords were: **Aged, Human, exercise, exercise therapy, motor activity** We added this to the information in the appendix.  

- The study selection initially indicates that articles evaluating people aged 50 and over will be included, but the study is for the aged population. At another point in the methods, it appears that people aged 60 and over will be included. Please clarify this point.

**  Answer: **We apologize if this was misleading. We revised this aspect as follows: Types of participants We will include studies involving participants aged > 50, according to the WHO definition of "older adults" (mean age of 60 years ± SD, with a minimum age of 50).   And in the next section:   Firstly, we will meticulously assess each study's eligibility criteria to ascertain if they meet our age inclusion (mean age of 60 years, age range starting at 50 years) and exclusion criteria for specific conditions.  

- Regarding the outcome of “functional mobility,” it was unclear why the daily walking distance and the number of daily steps were included among the primary outcomes. These outcomes are more directly related to the level of physical activity and could be on the list of secondary outcomes or just included as moderator variables.

**  Answer: **We thank you for this comment. We discussed ist comprehensivly in our group and would like to keep this aspect like it is, as the level of functional mobility is related to walking capacity and daily mobility as described in our introduction..  

- The description of the analyses for the meta-analysis is presented in detail, as are the strategies for presenting the results

**Answer.** We thank you for this comment and changed the sections as follows: On page 8 We will systematically collect data from studies that provide both baseline assessments and at least one post-intervention assessment. For studies with multiple post-intervention assessments, we will select the assessment closest to the completion of the exercise intervention as the primary data point for the meta-analysis. This choice aims to provide a consistent benchmark across studies. Additionally, to capture longer-term outcomes and the sustainability of intervention effects, follow-up assessments will be analyzed and reported separately. These follow-up results will help describe the maintenance of effects over time and offer insights into the durability of the intervention benefits. The clinically meaningful timing of outcome assessments will be determined by the nature of the intervention program and the specific pathology being addressed. For instance, shorter intervention periods may be more appropriate for acute conditions, whereas chronic conditions might require longer durations for meaningful outcomes to emerge. Consequently, our review will include evaluations categorized as short-term (within the first six months of treatment) and intermediate-term (from six to 12 months of treatment). This stratification allows for a nuanced understanding of how different interventions perform across varying timelines. And on page 14 To assess the possibility of publication bias and other reporting biases, we will conduct tests for funnel plot asymmetry for any analyses that include more than 10 data points. This threshold is consistent with the recommendations by Higgins et al. (2022), ensuring adequate power and reliability in identifying asymmetry. The funnel plot asymmetry tests will be applied separately for each comparison within the network, provided there are sufficient data points for meaningful analysis. In addition, comparison-adjusted funnel plots will be constructed as part of the network meta-analysis to detect potential small-study effects, which may arise from studies with smaller sample sizes reporting disproportionately large intervention effects. These plots will aid in identifying and interpreting potential biases across the network structure of the meta-analysis. A further measure to ensure impartiality in our review process will be implemented should any of the authors be contributors to a study included in the meta-analysis. In such cases, the involved authors will recuse themselves from conducting the risk of bias assessment and from participating in any GRADE (Grading of Recommendations Assessment, Development, and Evaluation) assessments that utilize data from their study. This measure safeguards the objectivity and transparency of our evaluation process. On page 15 Our review will employ a network meta-analysis (NMA) to comprehensively compare multiple exercise interventions and their effects. This method extends traditional meta-analysis by incorporating both direct comparisons (interventions compared within randomized controlled trials) and indirect comparisons (interventions compared across trials via a shared comparator). The NMA framework allows us to estimate the relative effectiveness of multiple interventions, even if some have not been directly compared in primary studies. Initially, a standard pairwise meta-analysis will be conducted to evaluate the intervention effects of individual exercise interventions. This preliminary step will establish the efficacy of each intervention in isolation. Subsequently, a mixed treatment comparison will integrate evidence from both direct and indirect comparisons using a consistency model to estimate relative treatment effects. Transitivity will be assessed by examining the distribution of effect modifiers (e.g., baseline characteristics, study design features) across the included studies to ensure that the conditions required for valid indirect comparisons are met. A network plot will be generated to visually represent the direct connections between different interventions, providing a clear depiction of the evidence base. The NMA will also allow us to investigate the influence of various moderators, such as participant characteristics (e.g., age, gender, baseline fitness levels) and intervention-specific factors (e.g., exercise intensity, frequency, and duration). To address potential inconsistencies within the network, inconsistency models may be employed. These models incorporate study-level covariates to account for variations across studies. If significant inconsistency is detected, we will explore adjustments such as including additional covariates or revising the model structure. This iterative process aims to ensure the robustness and reliability of the network meta-analysis findings.
